# Synergistic Effect of Sorafenib and Radiation on Human Oral Carcinoma *in vivo*

**DOI:** 10.1038/srep15391

**Published:** 2015-10-21

**Authors:** Fei-Ting Hsu, Betty Chang, John Chun-Hao Chen, I-Tsang Chiang, Yu-Chang Liu, Wei-Kang Kwang, Jeng-Jong Hwang

**Affiliations:** 1 Department of Biomedical Imaging and Radiological Sciences, National Yang-Ming University, No. 155, Sec. 2, Li-Nong St., Bei-tou, Taipei 112, Taiwan; 2 Department of Radiation Oncology, Mackay Memorial Hospital, No. 45, Minsheng Rd., Tamsui District, New Taipei City 251, Taiwan; 3 Department of Radiation Oncology, National Yang-Ming University Hospital, I-Lan 260, Taiwan; 4 Department of Anatomical Pathology, Cheng-Hsin General Hospital, Taipei 112, Taiwan; 5 Biophotonics and Molecular Imaging Research Center, National Yang-Ming University, Taipei, Taiwan

## Abstract

Oral squamous cell carcinoma often causes bone invasion resulting in poor prognosis and affects the quality of life for patients. Herein, we combined radiation with sorafenib, to evaluate the combination effect on tumor progression and bone erosion in an *in situ* human OSCC-bearing mouse model. Treatment procedure were arranged as following groups: (a) normal (no tumor); (b) control (with tumor); (c) sorafenib (10 mg/kg/day); (d) radiation (single dose of 6 Gy); (e) pretreatment (sorafenib treatment for 3 days prior to radiation), and (f) concurrent treatment (sorafenib and radiation on the same day). The inhibition of tumor growth and expression level of p65 of NF-κB in tumor tissues were the most significant in the pretreatment group. EMSA and Western blot showed that DNA/NF-κB activity and the expressions of NF-κB-associated proteins were down-regulated. Notably, little to no damage in mandibles and zygomas of mice treated with combination of sorafenib and radiation was found by micro-CT imaging. In conclusion, sorafenib combined with radiation suppresses radiation-induced NF-κB activity and its downstream proteins, which contribute to radioresistance and tumorigenesis. Additionally, bone destruction is also diminished, suggesting that combination treatment could be a potential strategy against human OSCC.

Human oral squamous cell carcinoma (OSCC) has been reported to be associated with betel quid chewing, cigarette smoking and alcohol consumption, which are risk factors for cancer development^1^,^2^. The mortality of oral cancer is ranked the fourth in Taiwan^3^, and about 2% among all cancers worldwide. The major treatments for oral cancer are radiotherapy (RT), chemotherapy and surgery, but with poor prognosis^4^. The estimated survival rates of 1-, 5- and 10-year for all stages after diagnosis is 84%, 61% and 51%, respectively^5^. Among the established treatment for oral cancer, RT is currently the standard adjuvant form of treatment^6^. However, DNA damage induced by radiation results in an increase in NF-κB/DNA binding activity if the double strand breaks were not repaired^7^. NF-κB signaling pathway can be activated by chemotherapeutic agents and RT, respectively^8^,^9^, followed by the increased expressions of downstream effector proteins, such as cyclin D1, B-cell lymphoma 2 (Bcl-2), tumor necrosis factor (TNF-α), vascular endothelial growth factor (VEGF), X-linked inhibitor of apoptosis protein (XIAP), matrix metalloproteinase 9 (MMP-9), and cyclooxygenase-2 (COX-2), and results in the tumor proliferation, anti-apoptosis, invasiveness and radioresistance^9^. NF-κB also has been shown to play a role in homeostasis of the bone. Mice deficient in both subunits of NF-κB would fail to generate mature osteoclasts, suggesting the necessity of NF-κB for the development of osteoclasts^10^. The production of receptor activator of NF-κB ligand (RANKL) by OSCC may alter the tumor microenvironment to increase the osteoclastogenesis and mediate local bone invasion^11^. Interaction of RANKL with its receptor, RANK, could stimulate osteoclast precursors to differentiate into mature osteoclasts, leading to severe bone destruction. However, the binding of RANKL to RANK can be inhibited by osteoprotegerin (OPG). During the process of bone resorption, growth factors are secreted into the microenvironment to promote the proliferation of oral cancer cells, which in turn release bone resorbing factors for the production of RANKL^12^. Bone invasion of OSCC usually indicates advanced cancer stage and poor prognosis^13^, the capability of OSCC to invade the nearby bones may reduce the quality of life of patients^14^. Therefore, inhibition of NF-κB activation may be a potential therapeutic strategy for the treatment of OSCC with advantage to reduce the bone destruction simultaneously.

Sorafenib, a multikinase inhibitor, has been approved by FDA for the treatment of several types of cancers including renal cell carcinoma, hepatoma and colorectal carcinoma through inhibition of several pathways such as Ras-Raf-MEK-ERK, VEGF receptor (VEGFR), and platelet-derived growth factor receptor (PDGFR)^15^. Nevertheless, sorafenib alone has been reported to have a low level of anticancer capability such that sorafenib combined with other agents is suggested to achieve the better therapeutic outcome^16^,^17^. Our previous study shows that sorafenib enhances the treatment outcome of radiation *via* suppression of ERK/NF-κB signaling pathway in human SAS oral cancer cell line^18^. However, it is still ambiguous whether such combination is effective in reduction of bone destruction while increasing the therapeutic efficacy of human OSCC *in vivo*. Here we evaluated the therapeutic efficacy and the underlying mechanism of sorafenib combined with ionizing radiation in orthotopic human SAS oral carcinoma-bearing mouse model using multimodalities of molecular imaging.

## Results

### Sorafenib Combined with Radiation Inhibits Tumor Growth in Orthotopic OSCC-bearing Animal Model

**Figure 1:**
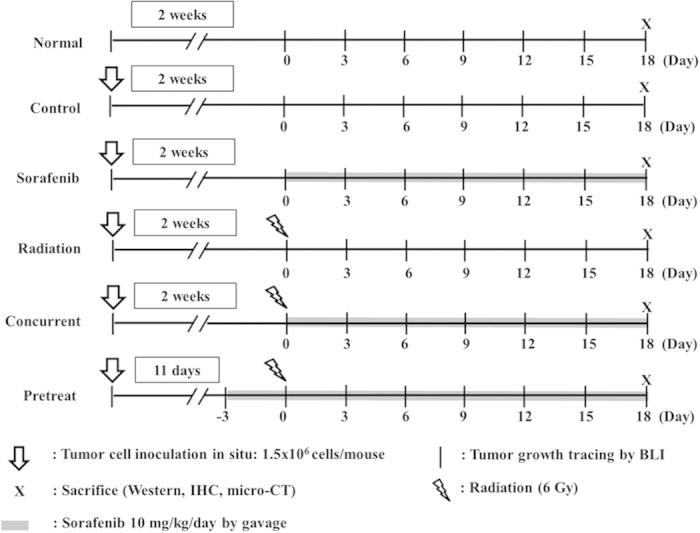
The experimental design for the treatment of human OSCC-bearing mice. Each mouse was injected with 1.5 × 10^6^ human oral squamous carcinoma SAS/*luc2* cells. Two weeks after tumor cell inoculation, mice were randomly divided into six groups (n = 10 per group). Sorafenib (10 mg/kg) was administered daily by gavage. For radiation treatment, mice were irradiated with single dose of 6 Gy on the head and neck region. All mice were sacrificed three weeks post treatments. The experiment was repeated three times.

**Figure 2:**
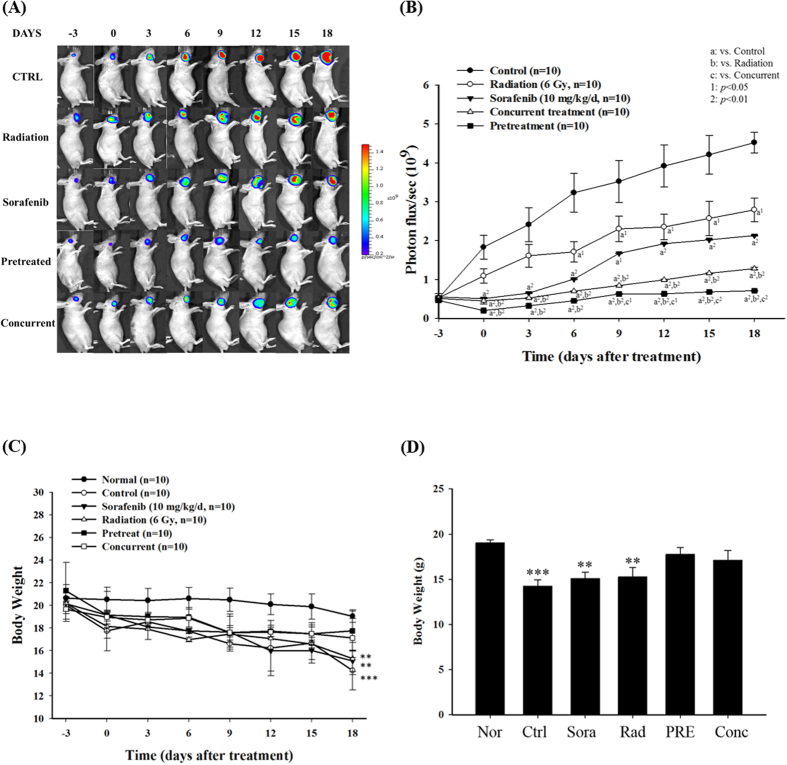
Therapeutic efficacy evaluation of sorafenib, radiation, and combination treatment of both on human OSCC-bearing mouse model. (**A**) Tumor growth monitoring was assayed by bioluminescent imaging (BLI). Either pretreatment or concurrent treatment significantly suppresses tumor growth compared to the other groups of mice (control, sorafenib and radiation). (**B**) The regions-of-interest (ROIs) of tumors as shown in (**A**) were quantified. Pretreatment of sorafenib combined with radiation shows the most prominent tumor inhibition. a^1^: *p* < 0.05, a^2^: *p* < 0.01 compared to that of the control; b^1^: *p* < 0.05, b^2^: *p* < 0.01 compared to that of radiation group; c^1^: *p* < 0.05, c^2^: *p* < 0.01 compared to that of the concurrent treatment. (**C**) The body weights of mice treated with combination of sorafenib and radiation remain similar to that of normal mice throughout the study period. However, the body weights of the control and irradiated mice drop significantly. ^***^p < 0.001 compared to that of the normal. (**D**) The body weight was measured by digital platform at day 18. ^***^p < 0.001 compared to that of the control. ^##^*p* < 0.01, ^###^*p* < 0.001 compared to that of the normal.

Orthotopic tumor-bearing nude mice were established by injecting 1.5 × 10^6^ SAS/*luc2* cells into the right masseter region of 6-week-old male mice. Two weeks later, mice were randomly divided into six groups as described in MATERIALS & METHODS and depicted in Fig. 1. BLI was used to evaluate the therapeutic efficacy of the treatments. As shown in Fig. 2A,B, photons emitted from the tumors of the combination group were significantly lower than those of the single treatment and the control groups, suggesting sorafenib could sensitize tumors to radiation therapy. The body weight was monitored from day −3 to day 18. Notably, no significant difference of body weight change among groups of pretreat, concurrent and the normal was found, indicating that both pretreatment and concurrent treatment of sorafenib combined with radiation were effective for the tumor control (Fig. 2C,D).

### Combination Treatment of Sorafenib and Radiation Inhibits Activation of NF-κB and Expressions of Its Downstream Effector Proteins

**Figure 3:**
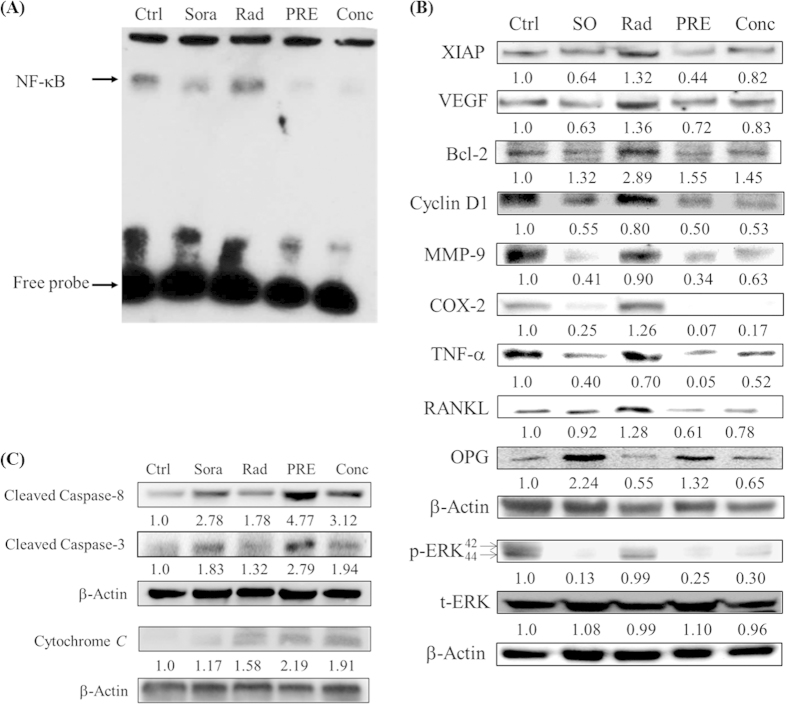
The effects of sorafenib and radiation on NF-κB activity and expressions of its downstream effector proteins in tumor tissues of SAS/*luc*-bearing mice. (**A**) The NF-κB /DNA binding activity was measured by electrophoretic mobility shift assay (EMSA) using nuclear extracts from tumor tissues of mice. The NF-κB/DNA binding activities are significantly suppressed by sorafenib alone, pre- and concurrent treatments, respectively, compared to those of the control and radiation. (**B**) Protein lysates extracted from tumor tissues of mice were assayed with Western blot. Expressions of radiation-induced NF-κB downstream effector proteins, such as XIAP, VEGF, COX-2 and RANKL, are significantly suppressed by pre- and concurrent treatments of sorafenib with radiation. Bcl-2, cyclin D1, MMP-9, TNF-α and p-EPK are also suppressed. (**C**) Expression levels of pro-apoptotic proteins (cleaved caspase-8 and cleaved caspase-3) induced by radiation were further increased in the groups of pre- and concurrent treatments. β-Actin was used as the internal control. Ctrl: Control, SO: sorafenib, Rad: radiation, PRE: pretreatment, Conc: concurrent treatment.

NF-κB plays a critical role in the regulation of proteins involved in the cell survival (Bcl-2, XIAP), proliferation (cyclin-D1), invasion (MMP-9, RANKL), angiogenesis (VEGF), and inflammation (COX-2, TNF-α), all contribute to the tumor progression. The activation of NF-κB could be determined by its binding activity with DNA using EMSA assay. As shown in Fig. 3A, sorafenib suppresses the radiation-induced NF-κB/DNA binding activity in both groups of mice with pretreatment and concurrent treatment, respectively. The expressions of NF-κB effector proteins were assayed with Western blot. Expressions of NF-κB–associated proteins induced by radiation were significantly reduced by sorafenib as shown in Fig. 3B. The inhibition of protein expressions related to the cell proliferation (cyclin D1) and tumor invasion (MMP-9 and RANKL) was more severe in the pretreatment group compared with that of the concurrent treatment group. The expressions of anti-apoptotic proteins such as Bcl-2 and XIAP were also suppressed (Fig. 3B), while the expressions of pro-apoptotic proteins involved in the mitochondria-dependent, and -independent apoptotic pathways such as cytochrome *C*, cleaved caspase-3 and cleaved caspase-8 were up-regulated (Fig. 3C).

### Combination of Sorafenib with Radiation Reduces Bone Invasion via Inhibition of Osteoclastogenesis

**Figure 4:**
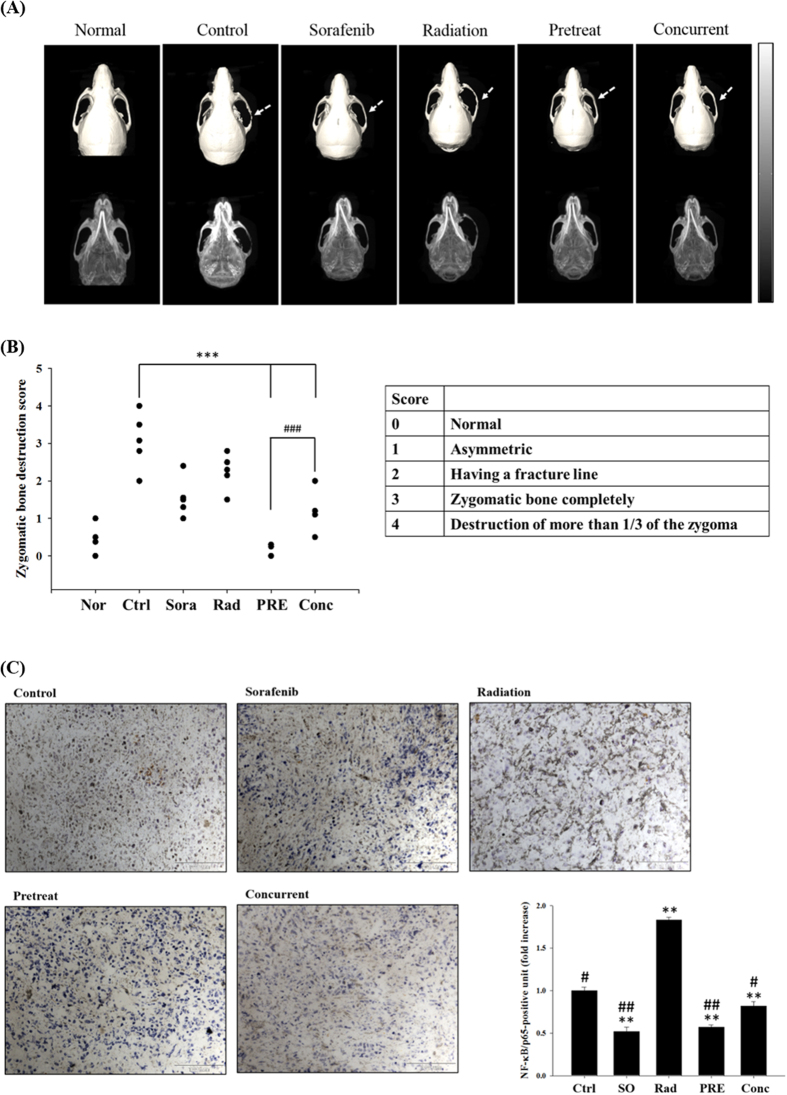
Zygomatic bone destruction in SAS/*luc*-bearing mouse model. (**A**) Zygomatic bone destruction in SAS/*luc*-bearing mouse model was assayed using micro-CT on day 32 post inoculation of tumor cells. The top row shows the top views of three-dimensional zygomatic bone images of mice, while the bottom row shows the bottom views of two-dimensional zygomatic bone images of the same mice. The zygomatic bone damage in the control mouse is demonstrated (pointed by an arrow). Notably, no or little damage to mandible and zygoma were found in mice treated with sorafenib combined with radiation, especially in mice of the pretreatment group. (**B**) The severity of zygomatic bone destruction was scored by five independent researchers. ^***^p < 0.001 compared to that of the DMSO-treated control, and ^###^*p* < 0.001 compared between the pre- and concurrent treatments. (**C**) Tumors from each group of mice were removed and sectioned for immunohistostaining of p65, a subunit of NF-κB. Cells stained in brown indicate p65-positive. NF-κB induced by radiation can be significantly suppressed by combination treatment of sorafenib and radiation. Bar = 100 μm, magnification = 200 x. ^*^p < 0.05 and ^**^p < 0.01 compared to that of the normal. ^#^*p* < 0.05 and ^##^*p* < 0.01 compared to that of radiation group.

Osteoclastogenesis can be induced through the RANKL/RANK signaling pathway, and lead to bone damage subsequently. The expression of RANKL induced by radiation was suppressed by sorafenib as shown in Fig. 3B. To evaluate the therapeutic effect on bone destruction, micro-computed tomography was performed on day 21 to reconstruct the images of mice heads. Figure 4A shows the representatives of three-dimensional and two-dimensional images of the zygomatic bone damage of each group. The zygoma of untreated control mouse was seriously damaged (pointed by an arrow). The damage of zygomas were reduced in mice treated with sorafenib and radiation alone, respectively. Little or no visible damage of zygoma was observed in both groups of pretreat and concurrent, suggesting the bone invasion could be suppressed by combination treatment. The score of the bone damage was performed by five independent researchers in a blind manner (Fig. 4B). In addition, the expression level of p65 (a subunit of NF-κB) in the tumor tissue was examined by immunohistostaining and the representative image of each group was shown in Fig. 4C. NF-κB induced by radiation could be suppressed significantly by sorafenib.

## Discussion

Current treatment protocol for OSCC with conventional fractionated radiotherapy delivers a total of 66–70 Gy to the primary tumor and involved lymph nodes. However, radiation dose exceeding 72 Gy may lead to unacceptable rates of normal tissue injury^19^,^20^, which limits the option of dose-escalation to achieve superior tumor control. Sequential or concurrent administration of chemotherapeutic agents to radiotherapy, such as fluorouracil and cisplatin^21^, paclitaxel^22^, docetaxel-cisplatin-fluorouracil^23^, carboplatin-fluorouracil^24^, is currently considered as the standard of care for advanced OSCC, which is classified as stage IV once mandibular bone or zygoma is invaded. Distant metastasis occurs in approximately 8–17% of patients who eventually die of the disease^25^. Hence, finding a strategy to reduce tumor growth and bone invasion is important for improving the treatment outcome.

The NF-κB signaling pathway in the cell can be activated by radiation *via* degradation of inhibitor |B protein (IκB). Activation of NF-κB subsequently leads to the expressions of proteins for pro-survival, anti-apoptosis, and results in the induction of radioresistance and tumorigenesis^26^. Constitutive activation of NF-κB in human head and neck squamous cell carcinoma is correlated to the resistance to chemo- or radiation therapy, which can be reduced through inhibition of NF-κB activation^27^. Sorafenib, a multi-kinase inhibitor, has been approved by FDA to treat human renal carcinoma and hepatoma. In our previous study, we have shown that sorafenib could suppress radiation-induced NF-κB activity and the expressions of its downstream effector proteins in a human colorectal carcinoma-bearing animal model^28^. Synergistic effect of sorafenib combined with radiation on human OSCC *in vitro* also has been shown^18^. Furthermore, NF-κB could be suppressed by sorafenib through the inhibition of RAF/MEK/ERK pathway^29^. Here we demonstrate that sorafenib can sensitize human OSCC to radiation and suppress the expressions of carcinogenic proteins through the inhibition of NF-κB *in vivo* (Fig. 3A). Although receptor tyrosine kinase inhibitors have been reported to be able to increase the level of COX-2, and lead to the development of drug resistance^30^,^31^, our results show that the expression of COX-2 in human OSCC can be suppressed by sorafenib *in vivo*. Furthermore, radiation-induced COX-2 expression also can be suppressed by sorafenib (Fig. 3B).

**Figure 5:**
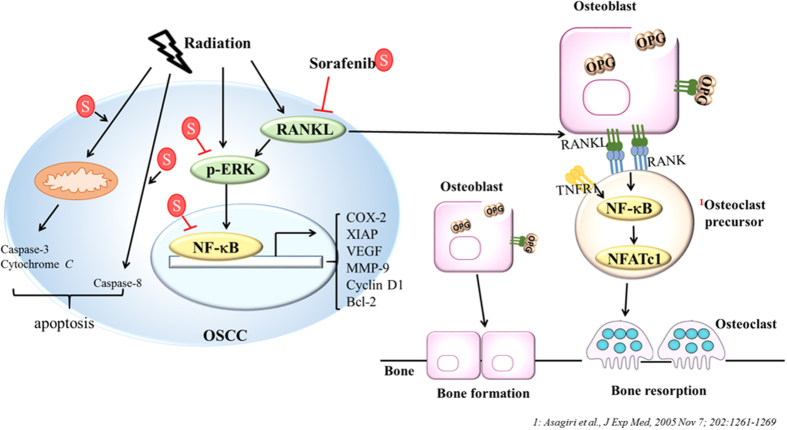
Schematic diagram depicting the signaling pathways involved in enhanced therapeutic efficacy and reduced bone erosion by combination treatment of sorafenib with ionizing radiation. Radiation induces cancer cell killing *via* enhancing the expressions of cytochrome *C* and cleaved caspases 3 and 8, so does sorafenib. Radiation also induces expressions of RANKL and ERK/NF-κB and results in expressions of anti-apoptotic proteins, such as Bcl-2 and XIAP. RANKL released from OSCC cancer cells binds to RANK on the surface of osteoclast precursor to produce NF-κB and NFATc1, and differentiates into osteoclast. These pathways, however, can be inhibited by sorafenib. S: sorafenib; NFATc1: Nuclear factor of activated T-cells, cytoplasmic 1.

NF-κB may also play an important role in bone destruction by cancer cells. A SCCVII, derived from a mouse OSCC cell line, bearing animal model used by Furuta *et al*. showed that zygomatic bone destruction was significantly suppressed by NBD peptide, a selective NF-κB inhibitor, as determined with micro-CT^32^. Interestingly, the bone damage in SAS/*luc2*-bearing mouse model observed in this study is less severe compared to that of the SCCVII animal model. This difference may due to the invasive capability that the SCCVII cancer cell is more invasive. In addition, cancer type and animal model may also contribute to the difference in bone invasiveness. The bones located in the vicinity of or surrounded by OSCC are often invaded by cancer cells, and results in osteoclastogenesis and bone damage for patients^33^. Preosteoclasts differentiate into mature osteoclasts *via* binding of RANKL to RANK, and lead to bone destruction to provide nutrients for the surrounding cancer cells to proliferate and grow. Oral cancer cells can release cytokines such as interleukin-6 (IL-6) and parathyroid hormone-related peptide (PTHrP) to induce osteoclastogenesis and stimulate the production of RANKL^34^. All three isoforms of RANKL (isoforms 1, 2, and 3) have been reported to be expressed in several OSCC cell lines. OSCC cells secrete pro-osteoclastogenic RANKL, which contributes to bone invasion and eventually causes bone destruction^11^. Here we found that the expression of RANKL isoform 2, a soluble form secreted into the cytoplasm, in the tumor was suppressed in OSCC-bearing mice treated with sorafenib prior to or concomitant with radiation (Fig. 3B). Furthermore, reconstruction of zygomatic bone images obtained from micro-CT show little or no bone damage in mice treated with combination of sorafenib and radiation (Fig. 4A,B). Therapeutic effect of sorafenib for the recurrent or metastatic squamous cell carcinoma of head-and-neck or nasopharyngeal carcinoma in a phase II clinical trial has been studied with unsatisfactory outcome^35^. Here we suggest that therapeutic effect of sorafenib combined with radiation against human oral carcinoma could be through the inhibition of RANKL/p-ERK/NF-κB pathway and the reduction of NF-κB regulated downstream effector proteins (Fig. 5). Additionally, our results also suggest that bone destruction caused by tumor invasion in human OSCC is through RANKL-RANK/NF-κB/NFATc1 pathway^36^, which can be suppressed by sorafenib through releasing OPG from osteoblasts to suppress RANKL binding to RANK (Fig. 5). These findings may have the potential application for treating patients with OSCC in clinic.

## Methods

### Establishment of SAS/*luc2* Cell Line and Cell Culture

The human oral squamous cell carcinoma, SAS, cell line was kindly provided by professor Kuo-Wei Chang of the Department of Dentistry at National Yang-Ming University, Taipei, Taiwan. The SAS cell line was transfected with a vector using CMV promoter to drive luciferase-2 reporter gene and renamed as SAS*/luc2*, and maintained in Dulbecco’s modified Eagles’ medium (DMEM) supplemented with 5% penicillin/streptomycin (Gibco®, Grand Island, NY, USA), and 10% fetal bovine serum (FBS; Hyclone) at 37 °C with 5% CO_2_. G418 (500 μg/mL; Calbiochem, Darmstadt, Germany) was added to the medium to stabilize SAS/*luc2* cell line.

### Animals

The experimental procedures of the animal study were performed in accordance to the protocols approved by the Animal Care and Use Committee at National Yang-Ming University. Six-weeks-old male BALB/cAnN.Cg-Foxn1nu/CrlNarl nude mice were purchased from the National Laboratory Animal Center (Taiwan) and housed in a pathogen-free animal facility. The animals were fed sterilized mouse chow and water.

### Plasmid transfection and Selection of Stable Clones

The transfection of SAS cells was performed using jetPEI^TM^ (Polyplus Transfection, Strasbourg, France). SAS cells (2 × 10^6^) were seeded in a 10-cm dish and allowed to grow for 24 h. The p-CMV-*luc2* vector (8 μg) and 16 μL of jetPEI^TM^ solution were diluted with 500 μL and 484 μL of 145 mM NaCl. The mixture was mixed evenly, and incubated at room temperature for 30 min. 1000 μL jetPEI^TM^/DNA mixture was then added to the SAS cells and incubated at 37 °C for 24 h. Cells were trypsinized and cultured with DMEM supplemented with 1 mg/mL G418, 1% penicillin/streptomycin, and 10% FBS for 2 weeks. The survived clones were isolated and assayed with bioluminescent imaging (BLI), and renamed as SAS/*luc2* cell line.

### Extraction of Sorfenib from Nexavar tablets

The extraction of sorafenib from Nexavar tablet (Bayer Healthcare Co, USA) were conducted as mentioned previously^28^.

### Orthotopic Human Oral Carcinoma-bearing Mouse Model

All male Balb/c nude mice were anesthetized with pentobarbital (50 mg/kg/mouse). 1.5 × 10^6^ SAS/*luc2* cells were prepared in PBS, and injected into the right submucosal masseter of the mouse with a 29-gauge hypodermic needle. Mice were then randomly divided into six groups (n = 10/group): (a) normal; (b) control (0.1 ml PBS/mouse/d by gavage); (c) sorafenib alone [0.1 ml/mouse/d (10 mg/kg) by gavage], (d) radiation alone (single dose of 6 Gy on day 1), (e) pretreatment [0.1 ml/mouse/d of sorafenib (10 mg/kg) for three days prior to 6 Gy irradiation, and continued till the end of the experiment], (f) concurrent [0.1 ml/mouse/d sorafenib (10 mg/kg) and 6 Gy irradiation on day 1, and continued sorafenib till the end of the experiment]. Radiation treatment was performed on the head and neck, while the rest of the body was shielded with a lead plate with 5 cm thickness. Irradiation was conducted using an X-ray irradiator (RS 2000; Rad Source Technologies, Suwanee, GA, USA) at a dose rate of 1.03 Gy/min, 80 cm source-to-skin distance (SSD), and field size of 30 × 30 cm^2^.

### *Ex-vivo* Western Blot Analysis

Lysates (60 μg) were extracted from the tumor tissues of mice, and were separated on 8–12% sodium dodecyl sulfate polyacrylamide gel electrophoresis (SDS-PAGE) then transferred onto polyvinylidene difluroide membrane (PVDF, Millipore, USA). The membranes were blocked with 5% non-fat milk, followed by incubated with appropriate primary antibodies (anti-XIAP, anti-VEGF, anti-Bcl-2, anti-cyclin D1, anti-MMP-9, anti-COX-2, anti-TNF-α, anti-RANKL, anti-OPG, anti-t-ERK, anti-p-ERK, anti-cytochrome *C*, anti-caspase 3 and anti-caspase 8 all purchased from Millipore) at 4 °C overnight with gentle shaking. A secondary peroxidase-conjugated anti-rabbit or anti-mouse antibody was diluted at 1:1000, followed by incubation for 1 hour at room temperature. The membranes were subjected to an enhanced chemiluminescence system, and immunoreactive bands were captured on the photographic film. The Image J software (National Institutes of Health, Bethesda, USA) was used for quantification. The experiments were repeated three times.

### *Ex Vivo* Electrophoretic Mobility Shift Assay (EMSA)

Nuclear proteins were extracted from tumor tissues of mice using the nuclear extraction kit (Chemicon International, Temecula, CA, USA). The binding activity of NF-κB was analyzed with the EMSA kit (Thermo Fisher Scientific Inc., Rockford, IL, USA). The oligionucleotide sequence for NF-κB is AGTTGAGGGGACTTTCCCAGGC. The non-labeled fragment sequence is GCCTGGGAAAGTCCCCTCAACT. Nuclear extracts were incubated with the biotin-labeled DNA probe at 25 °C for 20 min. A 5% polyacrylamide gel was used to separate the DNA-protein complexes and free oligonucleotides. After separation, the gel was transferred to a nylon membrane and cross-linked with an UV light, then further incubated with streptavidin-horseradish peroxidase for 15 min, and detected by enhanced chemiluminescence probe (ECL, Thermo Scientific Pierce Protein Biology Products, USA). The experiments were repeated three times.

### Bioluminescent Imaging (BLI) In Vivo

Mice bearing SAS/*luc2* tumors of each group (n = 5) were injected intraperitoneally with 200 μL of 150 mg/kg D-luciferin in PBS, and anesthetized with 1–2% isoflurane 10 min before imaging. Mice were set onto the imaging platform and continuously exposed to 1–2% isoflurane throughout the acquisition time. The tumor growth was monitored with BLI using IVIS50 Imaging System (Xenogen, Alameda, CA, USA) twice a week for 3 weeks. The body weight of mice was monitored twice a week until the end of the study. The photons emitted from the tumor were assayed using IVIS50 Imaging System. The acquisition time was 30 sec. Regions of interest (ROIs) were drawn around the tumor and quantified with the Living Image software (Xenogen, Alameda, CA, USA) as photons/s/cm^2^/sr.

### Micro-computed Tomography

Five weeks after tumor injection, five mice from each group were sacrificed and scanned with micro-computed tomography (micro-CT, Triumph X-O CT system, Gamma Medica Inc., Northridge, CA, USA). Two-dimensional and three-dimensional reconstruction images of the mouse head were acquired. The score of zygomatic bone destruction from the three-dimensional reconstruction image was evaluated as follows: 0: normal, 1: asymmetric, 2: having a fracture line, 3: zygomatic bone completely separated, and 4: destruction of more than 1/3 of the zygoma^32^. The severity of zygoma bone destruction was assessed by five independent researchers in a blinded manner. The experiments were repeated twice.

### Immunohistochemistry of p65, Subunit of NF-κB

Sections of paraffin embedded tumor tissue on the glass slides obtained from each group were deparaffinized in xylene, and rehydrated with decreasing concentrations of ethanol. The slides were then incubated in 3% H_2_O_2_ for 10 min. After washing, the slides were blocked with 5% normal goat serum for 5 min in a tight container, followed by incubation with primary anti-rabbit p65 antibody in a dilution of 1:100 (Millipore, USA) at 4 °C overnight. Finally, slides were counterstained with hematoxylin. At least three slides from each group were studied.

### Statistical Analysis

All data were represented with the mean ± standard error. Student’s *t*-test was used for the comparison between two groups. Kaplan-Meier plotting was used for the survival analysis, and the data were compared using the log-rank test. Difference between the means was considered significant if *p* < 0.05 or less.

## Additional Information

**How to cite this article**: Hsu, F.-T. *et al.* Synergistic Effect of Sorafenib and Radiation on Human Oral Carcinoma *in vivo*. *Sci. Rep.* **5**, 15391; doi: 10.1038/srep15391 (2015).
